# Aspects of the Health Inspection Authority in the People’s Republic of China

**DOI:** 10.1186/s12889-015-1832-0

**Published:** 2015-05-20

**Authors:** Sha Ma, Gang Chen, B-K Tan

**Affiliations:** Department of Health Law and Health Inspection, School of Public Health, Fudan University, 130 Dong’an Road, Shanghai, 200032 China; Faculty of Health Sciences, Curtin University, Kent Street, Bentley, 6102 Western Australia; Armadale Health Service, 3056 Albany Highway, Mount Nasura, 6112 Western Australia

**Keywords:** Health inspection authority, Institutional functions, Manpower, Revenue, Building area, Equipment

## Abstract

**Background:**

In China, there was a pressing need to establish a governmental agency to oversee the organizations that provide public health and medical services. The Chinese Health Inspection Authority (HIA), a relatively independent organization functioning at each administrative level (provincial, municipal, and county), was mandated to conduct 11 health inspection functions to maintain efficient public health and medical services. These functions include issuing health permit, conducting health supervision and inspection, health testing and evaluation, case investigation, complaint handling, managing public health crisis, monitoring and safeguarding public health at major public events, enforcing supervision and inspection compliance, public health education, information management, and team training and management. Since the reform of the health inspection system by the Ministry of Health in 2000, the HIA underwent a series of changes and transitions. This study aimed to describe and assess the five factors that were considered to be important for meeting service delivery objectives of the HIA in the People’s Republic of China.

**Methods:**

A total of 604 HIAs, sampled across three geographical regions of China at three administrative levels, participated in a cross-sectional survey conducted in 2013. Descriptive statistics were used to analyze the status of mandated operations, manpower, revenue and expenditures, and institutional infrastructure. Differences in these characteristics across the geographical regions and administrative levels were compared.

**Results:**

On average, the HIAs had not fully implemented the 11 mandated functions at any administrative levels. Governmental financial allocations were the main sources of revenue. Three primary personnel employment models coexisted and most employed the quasi-civil service employment model. The institutional infrastructure did not meet governmental mandated standards with respect to building area or the number and types of equipment available to conduct key functions.

**Conclusions:**

In 2012, the majority of the HIAs in China at the provincial, municipal, and county levels did not meet the mandated requirements, although positive indications toward meeting these requirements were observed. It is necessary for the government to pay more attention to institutional resources (buildings, equipment, and the level of the staff’s educational attainment) and ensure that the HIAs can meet their service delivery objectives.

## Background

### Health inspection in China

Health inspection has become more important to the public in mainland China as its awareness of preventive healthcare has grown [1]. In China, health inspection [2–5], means “health regulation,” which is a specific term for the governmental management of national public health affairs to maintain the orderliness and quality of public health and medical services for the protection of the people’s health [6]. The Health Inspection Authority (HIA) is a specific governmental organization within the Health Inspection Institution (HII) that was established to meet and enforce mandated health inspection functions. The health inspectors of the HIA and other organizations or individual contractors authorized by laws and regulations, are responsible for all of the activities of health inspection. Their authorization derives from a series of laws and regulations such as Law of the People’s Republic of China on the Prevention and Treatment of Infectious Diseases, Law of the People’s Republic of China on Prevention and Control of Occupational Diseases, Law of the People’s Republic of China on Medical Practitioners, Law of the People’s Republic of China on Blood Donation, Regulation on Hygienic Management in Public Venues, and Regulation on Medical Institutions. When specific laws and regulations are violated, or when there are threats to people’s health that originate from public health activities, these officials and staff investigate matters of legal liability.

### History and function of the Health Inspection Institution in China

Almost 30 years after establishment of the People’s Republic of China in 1949, the Epidemic Prevention Station (EPS) took responsibility for the administrative management, including the provision of technical services and developing governance, for China’s public health. This EPS was abolished during the Cultural Revolution [7, 8]. From then until 1995, the Food Hygiene Law [9], the legal forerunner of today’s health inspection system, mandated health inspection as a responsibility of the Ministry of Health (MoH). In the early 1990s, the HIA gradually emerged nationwide to attend to a myriad of the health inspection issues and provide chargeable technical services and advice. For example, part of the original EPS charged hospitals, food preparation producers, and other health facilities or individuals for services rendered.

**Fig. 1:**
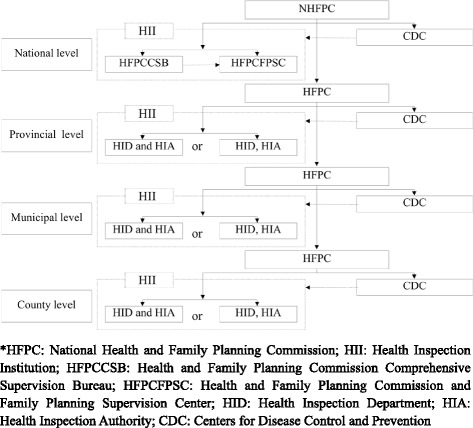
The health inspection system in the People’s Republic of China

In 2000, the MoH issued the Opinions on Health Inspection System Reform [10], which commenced the government’s formal reforms of the health inspection system. In those reforms, the original functions of the EPS were reintroduced and reorganized into the Chinese Center for Disease Control and Prevention (CDC) and the HII. These two organizations fall under the administrative jurisdiction of the National Health and Family Planning Commission (NHFPC), which replaced the MoH in 2012. The development and progress of the CDC was comprehensively documented by Hipgrave in 2011 [8]. At the national level, the HII has two branches - the Health and Family Planning Commission Comprehensive Supervision Bureau (HFPCCSB) and Health and Family Planning Commission and Family Planning Supervision Center HFPCFPSC (Fig. 1). At the provincial, municipal and county levels, the HII was organizationally structured into the Health Inspection Department (HID) and the HIA. In some smaller provinces, municipalities and counties, HID and HIA are combined as one entity and report to the HFPC (Fig. 1).

The division of responsibilities of these HID and HIA have a complex operational framework and reporting chain. Whilst the HID and the HIA belong to the HFPC management on the organizational chart, they are operationally different. The HID is a department within the HFPC and its employees are civil servants who report and are accountable to the HFPC. The HIA on the other hand is a semi-independent agency that employs a combination of civil servants and independent contractors to conduct health inspection and ensure compliance with health laws.

The Opinions on the Health Inspection System Reform clearly required that the HII employs hierarchical management at the central, provincial, municipal, and county levels [11]. Thus, as Fig. 1 illustrates, the framework of the Chinese health inspection system consists of the HIIs at four administrative levels [12]. The HIA at all administrative levels is responsible for executing the health laws and regulations that concern the inspection of public health and medical services, specifically regarding issues of infectious disease control, quality of drinking water, health supervision of hospitals, schools, and public venues such as hospitality, food preparation, sports facilities, and entertainment facilities. At each administrative level, the 11 mandated functions were: (1) issuing health administrative permit, (2) health supervision and inspection, (3) health testing and evaluation, (4) case investigation, (5) complaint handling, (6) managing public health crisis (e.g. infectious disease), (7) monitoring and safeguarding public health at major public events, (8) enforcing supervision and inspection compliance, (9) public health education, (10) information management of health supervision, and (11) health supervision team training and management.

### New opportunities and challenges for the Health Inspection Authority

In response to the outbreak of Severe Acute Respiratory Syndrome (SARS) in 2002 and 2003, the Chinese government focused on strengthening her public healthcare system. The SARS outbreak provided China with a rare opportunity to reform and develop her health inspection system and, over the past decade, the number of HIA has gradually increased. Whilst new opportunities exist for reforming and strengthening her health inspection systems, cultural, historical, economic and a deeply complex reporting system are challenges to the reforms. For example, although the HIA is affiliated with the HFPC, it has independent financing. The HIA is legally responsible for enforcing the completion of tasks that fulfill the health inspection functions, but its power is limited to carrying out directives issued by the HFPC, which is not responsible for law enforcement [13]. Thus, the HIA faces restrictive barriers to becoming an independent regulator [14]. Although the health inspection system is still in development and transition, China has built a relatively independent HIA within it to oversee public health and medical services. Its primary functions are the protection of the people’s health, the promotion of social stability, and the advancement of national development. However, scant research has examined the status of China’s HIA across her different regions and administrative levels. For example, it is presently unknown whether the municipal institutional facilities have satisfied relevant legal and mandatory requirements imposed at the national level or whether the amount of equipment available at the county-level institutional facilities meets institutional needs. Therefore, the two objectives of this study were to: (1) describe and contrast the characteristics of the HIAs at the provincial, municipal, and county levels by region across China and (2) identify problems in the HIA and offer recommendations for resolving them. This study investigated the status of implementation of the HIA’s functions, manpower, revenue sources and expenditures, and building areas and equipment (infrastructure) available at the provincial, municipal, and county levels across China. The discussion considers the problems encountered by the HIA facilities and provides policy recommendations for China’s HIA.

## Methods

This study was commissioned by the Bureau of Food Safety and Health Inspection within the NHFPC. The overall aim was to investigate of the status of the HIA nationwide and to obtain recommendations for further growth and development of the HIA. Three administrative levels of the HIA were examined: provincial, municipal, and county. This study was undertaken by the Department of Health Law and Health Inspection, School of Public Health, Fudan University, Shanghai.

### Sampling design

The sampling frame was 32 provincial, 344 municipal and 2788 county-level HIAs from which a sample consisting of 660 HIAs (32 provincial, 148 municipal, and 480 county-level HIAs) was selected. The sample at the provincial level was drawn from census data. There were 32 provincial HIAs in the population, excluding Hong Kong Special Administrative Region, Macao Special Administrative Region, and Taiwan province.

The samples at the municipal and county levels were derived using a systematic sampling methodology [15]. The sampling frames consisted of 344 HIAs at the municipal level and 2788 HIAs at the county level. A total of 148 municipal and 480 county HIA were selected which constituted 43.0 % and 17.0 % of the populations of the HIAs, respectively. One provincial (3 %), eight municipal (5.4 %), and 47 county (9.8 %) HIAs declined to participate in the survey. Thus, the final sample consisted of 604 HIAs (31 provincial, 140 municipal, and 433 county HIAs). The response rate of the three samples was 91.5 %.

### Questionnaire

The questionnaire, with the intention to gather basic information on the HIAs at all administrative levels and across all different regions, was developed by the researchers (GC and SM) from the Department of Health Law and Health Inspection, School of Public Health, Fudan University, Shanghai.

This questionnaire went through several iterations based on input from experts and governmental officials in the field of health inspection. The questions were organized into 16 sections for the respondents at the provincial level. The municipal and county level questionnaires consisted of those 16 sections plus a supplementary section requesting information regarding strategies that had been implemented to improve the capacities of the HIA.

The 16 sections requested information on: (1) the physical building environment of the institution, (2) the final report on the construction and development of the institutional facility, (3) manpower, (4) revenue, (5) building area, (6) equipment available, (7) the facility’s capacity to manage and respond to public health crises (e.g. SARS), (8) the facility’s ability to monitor and safeguard public health at major public events, (9) mandated functions of the facility, (10) the health inspection administrative counterpart, (11) suggestions for the development of the HIA, (12) external supporting organizations engaged in health inspection, (13) number of health inspection assistants, (14) any external audits conducted, (15) indicators of evaluation of the HIA’s functions, and (16) identification of factors that may impact the effectiveness of the health inspection system (the respondents were informed that information in this section was not analyzed, but used to provide a foundation for future studies).

This cross-sectional survey was administered between February 5 and February 28, 2013. An official letter was sent to all chairpersons of the HIAs that participated in the study. The document explained the purposes of the survey and invited the relevant directors or heads of the relevant departments to provide the information on the specific aspects of the HIAs for which they were assumed to have expert knowledge. For example, the directors of the finance department completed the section of the questionnaire related to finance (i.e. the section on revenue) and the directors of the personnel departments provided information related to manpower. The chairpersons of the HIA were the main contact persons and were responsible for ensuring that the appropriate and relevant persons completed the questionnaire. This study was reviewed and approved by the ethics board of Fudan University, which owns the data for formal reporting to the government and for scientific study.

### Quality control

Confidence in the accuracy of the data was vital to validity of the results. To assure data quality, the respondents were given written instructed on how to complete the questionnaire. The questionnaire was deliberately organized into thematic sections (e.g. personnel or finance) so that the respondents could easily respond to the questions in their areas of responsibility. Each respondent was provided with a compact disk with the instructions and questions. In addition, the contact information of a researcher assistant was provided and she was accessible by telephone to answer all of the respondents’ questions during the data collection phase. All submitted questionnaires were visually scanned before data entry and those which had multiple items missing data were omitted from the analysis.

### Measures

To address the specific aims of this study, the analysis used 13 variables selected from the following five sections of the questionnaire: (1) mandated functions of the facility, (2) manpower, (3) revenue, (4) building area, and (5) equipment available. Furthermore, it employed an indicator of the regional location of each institutional facility. A comprehensive report presenting all of the variables will be submitted to the governmental body that commissioned this study.

### Mandated functions

#### Responsibilities

The director or chairperson of each HIA at each administrative level answered questions regarding: (1) whether the functions such as food production, food transport, catering, comprehensive coordination, employers’ activities, occupational diseases’ prevention agency, and technical services agency had changed and (2) which specific administrative agency was responsible for the health inspection functions. With respect to administrative responsibilities, the response options were: (a) HID, (b) HIA, or (c) other agencies (OA).

#### Institutional functions

There were 11 health inspection functions presented in the questionnaire (as described above). The respondents were asked a series of questions regarding each function. For example, ‘Is this item the responsibility of this institution?’ The response options were either ‘yes’ or ‘no’. The number of ‘yes’ responses was used to calculate each institutional facility’s percentage of its responsibility for the 11 functions.

### Manpower

#### Responsibilities

The directors of the personnel departments provided information about their staff on: (1) the total number of the staff at the institutional facility, (2) each staff member’s age, (3) each staff member’s educational level, and (4) the institutional facility’s personnel employment model.

#### Total number of staff

The respondents were asked, ‘How many staff members are currently working at this institutional facility?’ Answers were numerical and open-ended at the end of the question.

#### Staff member’s age

The respondents were asked, ‘How old are the employees at this institutional facility?’ Respondents provided each staff member’s age on a spreadsheet.

##### Staff educational level

The respondents were asked to provide each staff member’s educational level. The response options were: (1) master’s degree, (2) university degree of 4 or 5 years, (3) college degree of 3 years, (4) high school degree, or (5) junior high school education.

##### Mandated educational standards

According to the minimum educational level requirements, the percentages of staff with a college degree and above at the provincial, municipal, and county levels are mandated to be not less than 98 %, 95 %, and 80 %, respectively [16]. These standards at the different administrative levels were used in the analysis to assess the extent to which the institutional facilities at the different administrative levels met the governmental standard requirements.

##### Personnel employment model

The respondents were asked about the type of personnel employment model adopted by their institutional facilities. For example, ‘which personnel employment model is used at this institutional facility?’ The respondents had the options to choose: (1) civil service, (2) quasi-civil service, (3) public institution, or (4) other model. The ‘civil service personnel employment model’ indicates that all employees at the HIA were civil servants who enjoyed all the benefits of that status, including the iron-rice bowl and social welfare after retirement. HIAs which employ this employment model are fully funded by the government. The ‘quasi-civil service model’ is similar to the civil service model in that it is funded by the government, however, the employees are not classified as civil servants, although they enjoy the same entitlements as civil servants. The ‘public institution model’ is a particular personnel employment model adopted among institutions in China focused on promoting social public welfare. Revenue sources come in part from the government and in part from the charging for services rendered to the public or organizations. These institutions therefore are similar to corporate organizations because they are responsible for their own financial viability and accountability. Information regarding personnel employment model is important in this study because it may affect the revenue source of the HIA. For example, if a HIA employs the civil service or quasi-civil service employment model, all relevant activities are funded by the government so the facilities can conduct all the mandated functions. On the other hand, if a HIA employs the public institution employment model, there may be potential for abuse of authority (for example, providing unwarranted services for profit).

### Revenue

#### Responsibilities

The directors of the finance departments answered the questions regarding their institutional facilities’ sources of income between 2007 and 2012.

#### Revenue source

The response options regarding sources of revenue were: (1) governmental allocations, (2) subsidies from the superior, and (3) other sources (including charges for services rendered). ‘Governmental allocations’ refers to a variety of fiscal allocations from the central government. ‘Subsidies from the superior’ is non-fiscal revenues provided by the Public Health Administrative Department for special work, such as scientific research funding, training expenses, or technical assistance costs incurred by the institutional facilities. ‘Other sources’ includes income that are not included in the above categories such as issuing a health administrative permits, fines, monitoring, training, and bank interest earned on deposits

#### Expenditures

Personnel expenditures and non-personnel expenditures were two types of financial expenditures by the HIAs that were included in this study. ‘Personnel expenditures’ means wages, allowances, bonuses, and social security payments to or on behalf of the personnel. ‘Non-personnel expenditures’ means the daily operating expenses of the institutional facilities.

### Building area

#### Responsibilities

The directors of the HIA offices provided the information on the total areas (in square meters) of their physical buildings in response to the question, ‘How large is the building area of this institutional facility?’

#### Mandated building area standards

Based on the NHFPC recommendations, adequate building size is an indicator of an HIA’s ability to perform its mandated functions. According to the building area mandated standard, each HIA space must be not less than 40 m^2^ per employee. HIAs with few personnel are required to have total building areas no smaller than 4800 m^2^, 2400 m^2^, and 1200 m^2^ at the provincial, municipal, and county levels, respectively [11]. These standards of building areas at the administrative levels were used in the analysis to assess the extent to which the HIAs at the different administrative levels met the governmental standard requirements.

### Equipment

#### Responsibilities

The directors of the HIA offices were asked about the equipment available at their institutional facilities. Specifically, they were asked to provide the types and number of equipment physically present at their sites. The types of equipment were with respect to: (1) environmental or school health (e.g. capnograph and formaldehyde analyzer), (2) prevention of infectious diseases and medical institutions, (3) products related to public health, (4) radiological protection, (5) occupational health monitoring, (6) food safety, (7) forensics tools, (8) office functioning, (9) law enforcement vehicles, and (10) information technology (e.g. network equipment and server).

#### Mandated equipment standards

The government-mandated standard of the types and number of equipment at the HIAs was based on the Standards for the Equipment used by the Health Inspection Authority (2011 Edition) [17], which sets forth the minimum number of given types of equipment at each administrative level. There are 133 types of equipment required to be available at the provincial HIAs, 115 types of equipment required at the municipal HIAs, and a minimum of 90 types of equipment required at the county-level HIAs. In this study, the ratio of the number of types of equipment actually available to the mandated number of types of equipment was used to indicate the percentage of equipment at a given HIA. For example, if a HIA office manager at the municipal level reported that there were 75 types of equipment available, that HIA would be at 65 % compliance with the mandated standard (75/115 × 100 % = 65 %).

### Regions

The region in which an HIA was located was indicated using a categorical variable based on administrative divisions, geographical locations, and economic factors. There are three regions. The eastern region included the provinces or municipalities with high GDP (Beijing Municipality, Tianjin Municipality, Shanghai Municipality, Jiangsu Province, Zhejiang Province, Fujian Province, Shandong Province, and Guangdong Province). The central region included the provinces or municipalities with moderate GDP (Hebei Province, Shanxi Province, Liaoning Province, Jilin Province, Heilongjiang Province, Anhui Province, Jiangxi Province, Henan Province, Hubei Province, Hunan Province, and Hainan Province). The western region included the provinces or municipalities with low GDP (Inner Mongolia Autonomous Region, Chongqing Municipality, Sichuan Province, Guizhou Province, Yunnan Province, Tibet Autonomous Region, Shaanxi Province, Gansu Province, Qinghai Province, Ningxia Hui Autonomous Region, and Xinjiang Uyghur Autonomous Region).

### Data analysis

The data were analyzed using SPSS 18.0 statistical software. Descriptive statistics were generated for the 11 health inspection functions across the three administrative levels. Descriptive statistics on staff educational levels and the personnel employment models were generated at each administrative level and by region.

Comparative analyses assessed the differences between the government-mandated standards and the facts at the HIAs. For example, according to the official standard, the percentage of staff with a college degree and above at the provincial, municipal, and county levels is mandated to be not less than 98 %, 95 %, and 80 %, respectively [16]. Those mandated percentages were compared to the actual percentages of staff with a college degree and above as reported by the directors of the personnel departments. Other comparisons between the HIAs’ actual conditions and the government-mandated standards regarding, for example, building area or types of equipment were similarly evaluated.

## Results

### Mandated functions

The HIA respondents at each administrative level were asked to indicate if they executed the 11 mandated functions at their institutions and to state the name of the specific administrative agency under the HII (HIA, HID, or OA) that was responsible for each health inspection function. A summary of the percentages of the 11 mandated health inspection functions carried out at the different levels is presented in Table 1. Overall, at the provincial level, implementation percentages were higher than at the municipal or county levels. Case investigation was the most frequently reported function executed at the three administrative levels. Although more than 80 % of the functions were carried out at all the administrative levels, only case investigation achieved 100 % at the provincial level, suggesting that many health inspection institutions across the administrative levels did not achieve full compliance. Only about 65.4 % at the county level reported conducting the function of Health Testing and Evaluation.

**Table 1:** Summary of the percentage of the mandated 11 health inspection functions carried out at three administrative levels in 2012

Functions	Provincial	Municipal	County
Issuing health administrative permits	91.1	82.2	80.6
Health supervision and inspection	93.0	94.3	87.7
Health testing and evaluation	83.5	71.9	65.4
Case investigation	100.0	98.6	97.1
Complaint handling	98.8	93.1	91.2
Managing public health crises (e.g. Infectious disease)	95.9	88.2	86.3
Monitoring and safeguarding public health at major public events	95.1	93.6	89.7
Enforcing supervision and inspection compliance	96.7	95.9	94.6
Public health education	95.9	92.7	93.7
Information management of health supervision	88.3	86.7	85.7
Health supervision team training and management	93.1	89.3	89.2

Table 2 shows the distribution of responsibility for the 11 health inspection functions among the HID, HIA, and OA at the provincial, municipal, and county levels. For example, 100 % of the HII at the provincial level reported executing the function of case investigation (Table 1). This function was predominately carried out by the HIA (96.1 %) and a very small percentage was conducted by HID (3.9 %) (Table 2). As Table 2 shows, it is evident that the HIA was mainly responsible for carrying out 10 of the 11 health inspection functions (the exception was issuing health administrative permits), which seems to be fairly equally distributed among the HID, HIA, and OA and this was consistent across the administrative levels (Table 2). Of the 71.9 % at the municipal level that reported conducting health testing and evaluation (Table 1), about one-third (34.8 %) was conducted by OA, 60.2 % by HIA, and only a small percentage by HID. This pattern was also observed at the county level (Table 2).

**Table 2:** Summary of the percentage of responsibilities for the 11 functions among the Health Inspection Department (HID), Health Inspection Authority (HIA), and Other Agencies (OA) at three administrative levels in 2012 (*n* = 602)

Functions	Provincial			Municipal			County		
	HID	HIA	OA	HID	HIA	OA	HID	HIA	OA
Issuing health administrative permits	34.2	37.8	28.0	37.6	32.9	29.5	32.9	33.5	33.6
Health supervision and inspection	11.5	83.0	5.5	6.1	86.3	7.6	7.4	84.4	8.2
Health testing and evaluation	10.0	72.7	17.4	4.9	60.2	34.8	4.3	68.4	27.4
Case investigation	3.9	96.1	0.0	3.9	94.8	1.3	3.0	96.0	1.0
Complaint handling	0.0	99.2	0.8	4.5	93.5	2.1	3.3	94.4	2.3
Managing public health crises (e.g. Infectious disease)	27.1	63.5	9.4	20.5	70.1	9.4	22.7	69.6	7.7
Monitoring and safeguarding public health at major public events	13.1	77.4	9.5	12.3	75.2	12.6	13.5	79.0	7.5
Enforcing supervision and inspection compliance	5.0	95.0	0.0	7.0	92.5	0.6	4.8	94.0	1.2
Public health education	15.8	83.3	0.9	6.5	89.9	3.6	7.7	91.0	1.2
Information management of health supervision	1.7	97.0	1.3	2.7	94.2	3.1	3.6	93.2	3.1
Health supervision team training and management	6.4	84.3	9.2	8.9	86.4	4.8	8.5	85.1	6.4

### Staff’s educational level

Nationwide, the percentages of staff with a college degree and above at the provincial, municipal, and county levels were 94.3 %, 87.4 %, and 74.2 %, respectively (Table 3). Compared to the mandated standard of 98 %, 95 %, and 80 % at the provincial, municipal, and county levels, respectively, there was still a gap between the actual staff’s educational level and the mandated standard. Table 3 shows the percentages of staff with different educational levels at the administrative levels and regions.

**Table 3:** Summary of the percentages of staff with different educational levels at three administrative levels and regions in 2012 (*n* = 562)

Level	Region	Percentage of staff with college degree and above	Percentage of staff with less than a college degree
Provincial	Nationwide	94.3	5.7
	Eastern	95.7	4.3
	Central	93.7	6.3
	Western	93.6	6.4
Municipal	Nationwide	87.4	12.6
	Eastern	93.1	6.9
	Central	86.0	14.0
	Western	85.1	14.9
County	Nationwide	74.2	25.8
	Eastern	86.0	14.0
	Central	64.1	35.9
	Western	82.7	17.3

### Personnel employment model

Nationwide, a majority of the HIAs at the provincial (76.7 %) and municipal levels (71.5 %) employed the quasi-civil service personnel employment model (Table 4) and this was consistently observed across the three geographical regions. More than 50 % of the county HIAs employed the public institution employment model except in the western region (28 %), where about 70 % employed the quasi-civil service employment model (Table 4).

**Table 4:** Summary of the number of HIAs, percentage of the types of personnel employment models employed by the HIAs at three administrative levels and regions in 2012 (*n* = 591)

Level	Region	Types of personnel employment models			
		Civil service	quasi-civil service	Public institution	Other
Provincial	Nationwide	1 (3.3)	23 (76.7)	6 (20.0)	—
	Eastern	1 (12.5)	6 (75.0)	1 (12.5)	—
	Central	—	7 (63.6)	4 (36.4)	—
	Western	—	10 (90.9)	1 (9.1)	—
Municipal	Nationwide	4 (3.3)	88 (71.5)	31 (25.2)	—
	Eastern	1 (3.9)	19 (73.1)	6 (23.1)	—
	Central	—	25 (52.1)	23 (47.9)	—
	Western	3 (6.1)	44 (89.8)	2 (4.1)	—
County	Nationwide	8 (1.8)	174 (39.7)	252 (57.5)	4 (0.9)
	Eastern	5 (5.4)	39 (42.4)	48 (52.2)	—
	Central	—	19 (10.7)	157 (88.2)	2 (1.1)
	Western	3 (1.8)	116 (69.1)	47 (28.0)	2 (1.2)

### Revenue sources and expenditures

**Fig. 2:**
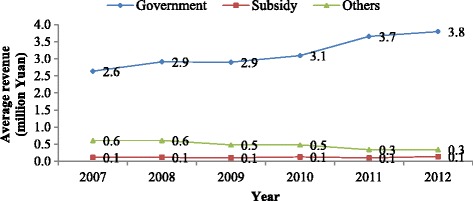
Changes in the distribution among revenue sources for HIAs nationwide: 2007–2012 (n = 604)

Figure 2 illustrates the three revenue sources (governmental allocations, subsidies, and other sources) from 2007 to 2012. Revenue from the government increased from about CNY 2.6 million per institutional facility in 2007 to about CNY 3.8 million per institutional facility in 2012. Revenue from other sources declined from about CNY 0.6 million per institutional facility in 2007 to about CNY 0.3 million per institutional facility in 2012. Revenue from subsidies remained consistent over that period.

The total revenue and expenditures in 2012 of the HIA at each administrative level and in each region are summarized in Table 5. Of the three revenue sources, governmental allocations were the main sources of revenue in 2012. Revenue from other sources (including income from services) at the provincial, municipal, and county levels accounted for only about 2.9 %, 6.9 %, and 9.1 % of total revenue, respectively (Table 5). The revenue in the eastern region was the highest and the western region reported the lowest, which was a consistent pattern across the administrative levels. Personnel expenditures were higher than non-personnel expenditures at all administrative levels (Table 5). Similar to the pattern found for revenue, the eastern region had the highest expenditures at the three administrative levels and the western region reported the lowest. The eastern-central-western pattern of declining expenditures was the same at the administrative levels.

**Table 5:** Distribution of the average revenue sources and expenditure for each HIAs at three administrative levels and regions in 2012 (*n* = 604) Million Yuan (%)

Level	Region	Revenue source				Expenditures		
		Total	Governmental allocation	Subsides	Other	Total	Personnel	Non-personnel
Provincial	Nationwide	29.5	28.2 (95.5)	0.4 (1.6)	1.1 (2.9)	23.5	10.9 (61.2)	7.9 (38.8)
	Eastern	14.6	13.9 (95.8)	0.9 (0.5)	0.4 (3.7)	11.1	6.3 (58.0)	3.4 (42.0)
	Central	12.0	11.5 (94.9)	0.6 (2.9)	0.3 (2.2)	9.9	5.6 (64.7)	3.6 (35.3)
	Western	18.0	17.2 (95.8)	0.7 (2.2)	0.6 (2.0)	13.4	7.1 (61.0)	4.5 (39.0)
Municipal	Nationwide	8.9	8.7 (91.8)	0.1 (1.3)	0.6 (6.9)	9.0	5.6 (73.3)	1.9 (26.7)
	Eastern	5.5	5.0 (94.1)	0.1 (0.7)	0.5 (5.2)	5.0	3.3 (74.9)	1.2 (25.1)
	Central	4.4	4.1 (91.1)	0.1 (1.4)	0.5 (7.4)	4.1	2.6 (72.9)	1.0 (27.1)
	Western	5.8	5.4 (90.3)	0.1 (1.6)	0.5 (8.1)	5.4	3.5 (72.2)	1.3 (27.8)
County	Nationwide	4.5	4.4 (88.4)	0.2 (2.6)	0.3 (9.1)	4.4	3.0 (69.2)	1.1 (30.8)
	Eastern	2.3	2.0 (91.7)	0.1 (2.8)	0.4 (5.4)	1.9	1.2 (72.9)	0.7 (27.1)
	Central	1.8	1.7 (82.4)	0.0* (3.1)	0.1 (14.5)	1.2	0.9 (65.3)	0.4 (34.7)
	Western	2.6	2.4 (91.9)	0.1 (1.4)	0.3 (6.7)	2.2	1.5 (68.6)	0.7 (31.4)

*less than 0.01 Million Yuan

### Building areas of HIA facilities

Table 6 presents a summary of the mean building areas, the mandated building areas, and the percentages of HIAs at the administrative levels and across regions that met the mandated building areas set by the government. Nationwide, less than 50 % of the HIAs met the requirements, with only 43 % compliance at the provincial level (Table 6). The eastern region HIAs had a highest percentage of compliance and the western region HIAs had a lowest compliance among the administrative levels (Table 6).

**Table 6:** Summary of the mean building areas, the mandated building areas and the percentage of HIAs that met the mandated standards across administrative levels and regions in 2012

Level	Region	Respondents	Met n (%)	Mean building area (m^2^)	Mandated standard (m^2^)
Provincial	Nationwide	28	12(42.9)	4543.8	4800
	Eastern	8	5(62.5)	6509.6	4800
	Central	11	4(36.4)	3946.9	4800
	Western	9	3(33.3)	3525.8	4800
Municipal	Nationwide	117	31(26.5)	1816.2	2400
	Eastern	26	10(38.5)	2312.1	2400
	Central	47	13(27.7)	1936.3	2400
	Western	44	8(18.2)	1394.9	2400
County	Nationwide	425	78(18.4)	727.8	1200
	Eastern	90	36(40.0)	1457.4	1200
	Central	173	27(15.6)	660.8	1200
	Western	162	15(9.3)	393.9	1200

A summary of the mean building areas per employee, the mandated building areas per employee, and the percentages of the HIAs that met the mandated standards at the administrative levels and across regions in 2012 is presented in Table 7. Similar to the patterns observed for the mean building areas, there was a higher percentage of HIAs that met the mandated building area per employee at the provincial level than at the municipal or county levels. In general, the eastern region had a higher mean building area per employee than the other regions (Table 7).

**Table 7:** Summary of the mean building areas per employee, the mandated building area per employee and the percentage of HIA that met the mandated standards across administrative levels and regions in 2012

Level	Region	Respondents	Met	Mean building area per employee (m^2^)	Mandated standards (m^2^)
		*n*	*n* (%)		
Provincial	Nationwide	28	18(64.3)	49.7	40
	Eastern	8	6(75.0)	65.7	40
	Central	11	5(45.5)	44.0	40
	Western	9	7(77.8)	40.6	40
Municipal	Nationwide	112	44(39.3)	38.3	40
	Eastern	25	14(56.0)	45.5	40
	Central	46	18(39.1)	36.2	40
	Western	41	12(29.3)	35.9	40
County	Nationwide	417	124(29.7)	32.2	40
	Eastern	90	43(47.8)	48.9	40
	Central	169	41(24.3)	24.2	40
	Western	158	40(25.3)	29.0	40

### Equipment available at HIA facilities

A summary of the mean numbers of equipment available, the mandated numbers of equipment set by the government, and the percentages of HIAs that met the requirements across the administrative levels and regions in 2012 are presented in Table 8. The disparity in the numbers of equipment available was most obvious at the provincial level where the eastern region reported the most types of equipment per institution (*n* = 69) and the central region reported the least (*n* = 30). In general, the HIAs at the provincial level had more types of equipment than the municipal or county HIAs. None of the HIAs reported 100 % of the types of equipment mandated by the government, suggesting that full compliance was generally not met. Overall, the percentages of the number of types of equipment available relative to the mandated requirements were low at all administrative levels across three regions (Table 8).

**Table 8:** Summary of the mean number of equipment available, the mandated number of equipment set by the government and the percentage of HIAs that met the requirements across administrative levels and regions in 2012

Level	Region	Mean number *n*	Mandated number *n*	Percentage %
Provincial	Nationwide	48	133	35.8
	Eastern	69	133	51.9
	Central	30	133	22.4
	Western	50	133	37.7
Municipal	Nationwide	33	115	29.0
	Eastern	46	115	40.2
	Central	30	115	26.4
	Western	28	115	24.7
County	Nationwide	25	90	27.7
	Eastern	28	90	31.0
	Central	23	90	26.0
	Western	25	90	27.9

## Discussion

HIA is China’s health law enforcement agency. The responsibilities of the HIA are to implement the 11 mandated functions stipulated by law to ensure the health and wellbeing of the Chinese in this populous country. The implementation of these mandated functions across China at the provincial, municipal, and county levels in the three geographical areas is difficult in such an expansive country and it is challenging to ensure that the HIAs adhere to the functions and requirements set by the government. Recent examples of poorly regulated food safety standards (e.g. the case of tainted milk powder in 2008) resulted in the people’s dissatisfaction and anger toward the health inspection standards in China. In 2008, when this problem occurred, three organizations (the MoH, the Administration of Quality Supervision, Inspection and Quarantine, and the Industrial and Commercial Bureau) were responsible for monitoring food safety standards. Poor coordination and collaboration among these organizations and a lack of clear boundaries regarding responsibilities and roles led to such cases slipping through the system. Since then, clearer roles and responsibilities have been implemented in the HIA to ensure that this problem is not repeated and to further to protect the health and safety of the Chinese people.

Since the reform in 2000, this is the second nationwide audit conducted to assess the status of the HIAs across China. Appropriate level of staff’s educational qualifications, adequate revenue sources, building areas, and equipment were assumed key factors for effective operation of the HIA, hence, the mandated minimum standards defined by the NHFPC. The assumption was that it is necessary to ensure that these key foundations are met if a HIA aims to implement all of its functions and to maintain its reputation.

This study found that, although most of the functions were conducted by the HIAs, some functions were conducted (and possibly duplicated) by the HID and OA. This is not surprising because China’s health inspection system underwent some adjustments. For example, health inspection functions regarding food health and occupational health underwent adjustments in response to policies of the health administrative department [18, 19]. The inconsistent pace of the transition created blurred boundaries regarding which agency was responsible for what function. Similar problems have been reported in the health institutions of other countries. For example, in some European Union countries, more than one authority were responsible for health inspection [20], resulting in a waste of resources.

The irregular implementation of the transition of functions across the three administrative levels, compounded by cultural, historical, and economic differences across the geographic regions, have created a deeply complex health inspection system. It is therefore crucial for the Chinese government to learn from other systems and develop a better clarity of the roles, responsibilities, and reporting lines among the relative administrative departments. Several countries, such as Chile and United States, have found that designing appropriate regulatory mechanisms is difficult and the actual enforcement of these mechanisms imposes high administrative costs on both the regulator (state) and the facilities that are being regulated [2]. A proposal emerging from this study is that China amalgamate its health inspection activities into one independent HIA at the provincial, municipal, and county levels.

The percentages of HIAs that had fully implemented the functions exceeded 80 % in 2012. Apart from health testing and evaluation, HIAs at the provincial level reported conducting more than 90 % of the mandated functions, higher than reported at the municipal or county levels. This is likely related to the relatively better-educated staff and adequate number of equipment at the provincial level.

The percentages of HIAs that conducted health testing and evaluation were noticeably low, particularly at the municipal and county levels. Health testing and evaluation require expert knowledge and sophisticated equipment [21] and are, therefore, more costly to execute than other functions. Some factors that contributed to low implementation likely include: (1) the relatively lower financial allocations from the government to the municipal and county HIAs, (2) less equipment available, and (3) the fewer staff with a college degree and above. Hence, the municipal and county HIAs had to depend on experts from the CDC or other special technical organizations [12], which would explain why almost one-third of the health testing and evaluation was reported in this study to be conducted by OA. The legality of OA conducting health testing and evaluation is however debatable. Health testing and evaluation has the potential to raise revenue and therefore may be open to abuse (e.g. bribery or issuing bogus health permits) if not well monitored and controlled.

Overall, the HIAs failed to meet the minimum educational requirement for staff at all administrative levels. A minimum educational level was mandated because health inspectors are required to have competency regarding health inspection laws and regulations and capability in specialized skills and knowledge [5]. This study found that there were gaps between the actual percentage of employees with a college degree and above and the mandated standards at all the administrative levels, but particularly at the municipal and county levels. It is therefore necessary for policies regarding employment requirements to be implemented as well as training and professional development opportunities for existing employees.

Theoretically, all HIAs nationwide should employ the same personnel employment model because their functions, roles, and responsibilities are regulated by national law. The HIA is the governmental organization responsible for health law enforcement, so its staff should be civil servants so that they can conduct their duties to implement the functions regulated by the law. This study found that three or more different employment models coexisted in the HIAs in 2012. This may be due to the incomplete reform of the health inspection system. During the reform, some HIAs refused to adopt the civil service employment model possibly because they believed that a change in employment model may lead to decreases in salaries. This may have resulted in a compromise – to adopt a ‘quasi-civil service’ employment model where HIAs are still fully funded by the government, and the staff, although lacking the prestigious title and status of civil servant, would enjoy the same entitlements of a civil servant. Compared with the results of the first audit in 2006 [5], this present study found that more HIAs reported using the quasi-civil service employment model and a lesser number of the HIAs used the public institution model. By the end of 2012, most of the HIAs employed the quasi-civil service model except at the county level.

An organization’s ability to operate is inseparable from its capital support. Health inspection in China is an aspect of the governmental management of healthcare that is distinct from general health services. From an economic standpoint, it is necessary for the institutional facility to acquire outright financial support from the government in the form of revenue because health inspection is a service to the public [22]. In the early 1980s, China’s financial decentralization was applied in the commercial and public sectors, leaving provincial and county governments mostly to fend for themselves [8]. With the reform still underway, this study found that the amount of revenue has gradually increased over recent years, with governmental allocations being the major sources of revenue. On average, HIA revenue in 2005 was CNY 10.5, 3.4, and 1.5 million at the provincial, municipal, and county levels, respectively [23]. In contrast, the mean revenue of HIAs in 2012 had increased almost threefold to CNY 29.5, 8.9, and 4.5 million at the provincial, municipal, and county levels, respectively.

A small and slowly declining revenue source was obtained from other sources such as fees for services (e.g. training and health inspection monitoring). Income generated from fees for services may result in abuses, such as bribery or issuing bogus health permits, which can potentially damage the reputation and credibility of the HIA. It is therefore important that the HIA move to gradually decrease the revenue derived from fees for services or other sources and increase the revenue from the government.

In addition to revenue and personnel concerns, infrastructure, as office buildings and equipment, is a key factor for the HIA to adequately perform its mandated functions [24]. Overall, the status of infrastructure at the three administrative levels paints a negative picture of the HIA. This study found that most HIAs had failed to comply at every level, particularly in the western region at the municipal and county levels. Each western HIA facility at the county level had only 393.9 m^2^, on average, perhaps because of lack of funds. Although the Chinese government has had a fiscal transfer payments policy in the western region for many years, the increased fiscal input is not commensurate with the output in these regions. To ensure that HIAs have adequate equipment and building areas, the HFPC and Ministry of Finance should not only increase the financial allocations to the HIAs, it should take action to ensure that the HIAs are accountable for their expenditures to meet the mandated standards.

This study has several limitations. First, the data collected was solely dependent on the respondents providing accurate data. Unfortunately, this cannot be verified despite efforts to ensure that the respondents for each of the sections were the persons with best knowledge to provide the information for that section. Second, the restriction of answers to ‘yes’ and ‘no’ in the questionnaires limited the study’s ability to gather nuanced, qualitative evaluation. Third, this is a cross-sectional survey and therefore, trends of change over time cannot be analyzed. Future studies should employ yearly monitoring data to assess trends and monitor the transition and the implementation of the health inspection reform.

## Conclusions

China’s health inspection system is in the midst of a transition. Factors such as manpower, revenue and infrastructure are vital to efficiently and effectively implement the functions of HIA. Ultimately, shortfalls in mandated standards directly or indirectly influence whether the HIA can achieve its purpose of protecting public health. The gaps between the actual conditions and mandated standards regarding staff educational level and infrastructure (building areas and the number of equipment types) are of the greatest concern in this regard. This study found these problems at the three administrative levels in every region, suggesting an urgent need for the government to implement policies to improve the capacity of the HIAs to carry out the mandated functions for the public health.

## References

[1] Zhu BD (1990). Discussion on the health supervision of China. Chinese J Public Health Management.

[2] Kumaranayake L (1997). The role of regulation: influencing private sector activity within health sector reform. J Int Dev.

[3] Stenson B, Syhakhang L, Lundborg CS, Eriksson B, Tomson G (2001). Private pharmacy and regulation - A randomized trial in Lao P.D.R. Pharmacy Practice and Regulation in Laos. Int J Technol Assess Health Care.

[4] Liu XG. Study on the problems of the Chinese health inspection system at the county level. Master’s thesis. Graduate School of National University of Defense Technology; 2006. http://cdmd.cnki.com.cn/Article/CDMD-90002-2007140787.htm, accessed 26 April 2014.

[5] Fan LH (2013). Health Supervision.

[6] Zhang FY, Liu N. Discussion on the status and strategies of the Chinese health inspection system. China Health Industry. 2013;38–39.

[7] Cui X (2007). The historical evolution of the Chinese health inspection system. Chinese J Health Inspection.

[8] Hipgrave D. Communicable disease control in China: from Mao to now. J Global Health. 2011;224–238. PMC3484775 23198121

[9] National People’s Congress. Law for Food Hygiene in the People’s Republic of China, 1 August 2005. 2005. http://www.gov.cn/banshi/2005-08/01/content_18960.htm, accessed 26 April 2014.

[10] Ministry of Health in the People’s Republic of China. The Opinions on Health Inspection System Reform, 19 January 2000. 2000. http://www.chinalawedu.com/falvfagui/fg22598/25524.shtml, accessed 26 April 2014.

[11] Ministry of Health in the People’s Republic of China. Provisions on the Construction of Health Inspection System in the People’s Republic of China (NO.39), 5 January 2005. 2005. http://www.moh.gov.cn/zhuzhan/wsbmgzl/200804/3c04780b0936408cbbe724abd2914733.shtml, accessed 26 April 2014.

[12] Zhao TG (2009). Progress and thinking to the China health inspection system reform and construction. Chinese Health Law.

[13] Ren WL (2004). The difficult and suggestion of the reform of the China health inspection system. Health Manag.

[14] Fang J (2008). The Chinese health care regulations in an era of transition. Soc Sci Med.

[15] Babbie E (2013). The practice of social research.

[16] National Center for Health Inspection and Supervision. Planning of education training for the national health inspector in 2005 to 2012, 20 March 2006.2006. http://www.jdzx.net.cn/article/402888830892e19c0108931795c20002/2009/3/402881e40a12a077010a1704553f0006.html, accessed 26 April 2014.

[17] Ministry of Health in the People’s Republic of China. Standards for the equipment used by the Health Inspection Institution (2011 Edition), 21 February 2012. 2012. http://www.sdwsjd.gov.cn/article/484848ac23eee6cf0123ef189d2b0007/2012/2/40288384341178b401359ea408400290.html, accessed 26 April 2014.

[18] Li JH (2005). Thinking on the food health inspection system’s adjustment. Chinese Public Health Manag.

[19] National Health and Family Planning Commission. Notice of the opinion on occupation health inspection management function, 17 January 2005. 2005. http://ziliao.aqsc.cn/law/zyws/102809/115531.html, accessed 26 April 2014.

[20] Varvara M, Sandra W, Gordon N, Tobias R, Mel S, Christopher B (2010). Hygiene inspections on passenger ships in Europe - an overview. BMC Public Health.

[21] Hou ZF (2006). Research and analysis on the status of Chinese health inspection. J Chinese Health Inspection.

[22] Zhu LC, Ma JF (2007). Thinking to the reform of the health inspection system. Occupation and Health.

[23] Zhang WH, Chen G, Li CY, Xu TQ, Zhang TX, Yang ML (2007). The status of finance revenue and expenditure of Health Inspection Authority in the People’s Republic of China. Chinese J Health Inspection.

[24] National Health and Family Planning Commission. Guiding opinions for the Health Inspection Authority’s construction, 9 February 2006. 2006. http://www.jdzx.net.cn/article/402881e4094442080109473d7ad00020/2009/3/402881e4094c8c0601094e6f69db000a.html, accessed 26 April 2014.

